# NEDD9 may regulate hepatocellular carcinoma cell metastasis by promoting epithelial-mesenchymal-transition and stemness via repressing Smad7

**DOI:** 10.18632/oncotarget.13852

**Published:** 2016-12-10

**Authors:** Zhipeng Wang, Min Shen, Peng Lu, Xiao Li, Shaojun Zhu, Shuqiang Yue

**Affiliations:** ^1^ School of Pharmacy, Fourth Military Medical University, Xi'an, China; ^2^ Department of Cardiovascular diseases, Xijing Hospital, Xi'an, China; ^3^ Department of Hepatobiliary Surgery, Hainan Branch of Chinese PLA general Hospital, Sanya, China; ^4^ Department of Hepatobiliary Surgery, Xijing Hospital, Xi'an, China; ^5^ Department of Pathology, Tangdu Hospital, Xian, China

**Keywords:** hepatocellular carcinoma, cancer stem cell, epithelial-mesenchymal-transition, developmentally downregulated 9

## Abstract

Overexpression of neural precursor cell expressed, developmentally downregulated 9 (NEDD9) is a prognostic marker of many cancers, including hepatocellular carcinoma (HCC). However, the functions and mechanisms of NEDD9 are unclear. We found that upregulation of NEDD9 promoted migration, invasion and cell-to-extracellular matrix adhesion of HCC cells. NEDD9 also induced the epithelial-mesenchymal transition (EMT) and expression of matrix metalloprotein 2 (MMP2). Increased aldehyde dehydrogenase (ALDH) activity and CD133-positive cells were observed in HCC cells with high expression of NEDD9, corresponding to greater sphere formation in cancer stem cells (CSCs). NEDD9 deregulated Smad7 expression to inhibit Smad signaling and binding to the FAK-Src-Crk complex. We propose that this is the mechanism by which NEDD9 induced CSC properties.

## INTRODUCTION

Metastasis is a key cause of poor long-term survival after surgical resection in hepatocellular carcinoma (HCC) patients, even as survival of patients with HCC improves [1]. Understanding the mechanisms of HCC metastasis will help develop better therapeutic strategies to improve the treatment of HCC and survival of HCC patients.

Increasing evidence suggests that aberrant activation of the epithelial-mesenchymal transition (EMT) triggers malignant tumor progression [2]. EMT is a complex biological process characterized by the loss of epithelial markers and acquisition of mesenchymal markers that are involved in cancer metastasis [15]. Cancer cells that undergo EMT lose epithelial adhesion characteristics, easily detach from primary sites, and invade the lymphatic or vascular systems [3, 4]. EMT also induces cells toward “stemness.” Cells with these properties are found in various cancer types. They are considered cancer stem-like cells (CSCs) [5] and are important in metastasis. Many signal pathways are involved in regulating EMT and CSCs including the pathways of Wnt, Notch, TGF-beta and NF-κB [6]. Defining the molecules that are critical to regulating EMT and CSC could lead to valuable strategies for HCC therapies.

The Smad signaling pathways are important in the EMT process [18]. Smad7 inhibits transforming growth factor (TGF)-β by binding to TGF-β receptor type I and blocking TGF-β-associated Smad signaling. Smad7 regulates the TGF-β1 signal pathway by inhibiting phosphorylation of Smad2,3 [19]. This process drives cancer progression and metastasis by promoting angiogenesis and inducing EMT [32]. Smad7 is highly expressed in cholangiocarcinoma and involved in cancer EMT and metastasis and is a potential prognostic cancer marker [20]. Smad7 also inhibits melanoma metastasis to bone [21]. Smad7 expression is deregulated in many cancers, suggesting that Smad7 might be a target for interfering with the development and progression of human cancers.

Nural precursor cell expressed, developmentally downregulated 9 (NEDD9), also known as Crk-associated substrate lymphocyte type (Cas-L) [7] or human enhancer of filamentation-1 (HEF1)[8], is a focal adhesion scaffold protein. NEDD9 is one of four members of a family of protein scaffold and adaptor molecules that are required for the assembly of signal transduction complexes in response to stimuli such as growth factors, hormones and extracellular matrix (ECM) components [22]. In normal tissues, levels of NEDD9 mRNA and protein are high in the lung, kidney, and fetal brain and in tissues rich in immature lymphoid cells [23]. NEDD9 expression is elevated in tumor cell lines derived from epithelial lineages such as glioblastomas and lymphomas [12, 24]. NEDD9 overexpression, as an oncogenic signaling abnormality, is associated with metastasis in several carcinomas, including glioblastomas, osteosarcomas and melanomas [25, 13]. Overexpression of NEDD9 protein correlates with poor prognosis in cancers including breast cancer, glioblastoma, lung cancer and melanoma [11, 12, 13]. We previously found NEDD9 was upregulated in HCC tissues and negatively correlates with overall survival. Zhang et al. also showed that NEDD9 protein levels were positively related to the invasion depth of gastric cancer and tumor lymph node metastasis [26]. Expression of NEDD9 is developmentally regulated in the early embryonic, but not adult, mouse brain [9]. NEDD9 has a crucial function in processes such as migration, mitosis, differentiation and apoptosis [10].

Izumchenko et al. studied the function of NEDD9 in mammary tumorigenesis using NEDD9 gene ablation in an MMTV-PyMT mouse model [33]. Loss of NEDD9 impairs mammary tumor development by limiting the activation of multiple pro-oncogenic signaling proteins, including the binding partners FAK and Src. Depletion of NEDD9 lead to defective cell spreading and migration and a greater susceptibility to anoikis due to downregulation of FAK activation [33].

In our previous study [14], HCC patients whose tumors had high NEDD9 expression showed early metastasis and poorer recurrence-free and overall survival than patients whose tumors had low NEDD9 expression. These results suggest that NEDD9 may be a valuable prognostic biomarker for HCC. However, the mechanisms by which NEDD9 influences HCC progression remain unclear. Identification of these mechanisms might be useful for developing treatments for HCC. This study aimed to identify the key function of NEDD9 in HCC metastasis. We identified the effect of NEDD9 on regulation of the Smad pathway and propose that NEDD9 induced EMT and increased stemness in HCC cells.

## RESULTS

### HCC with high NEDD9 expression underwent EMT

**Figure 1:**
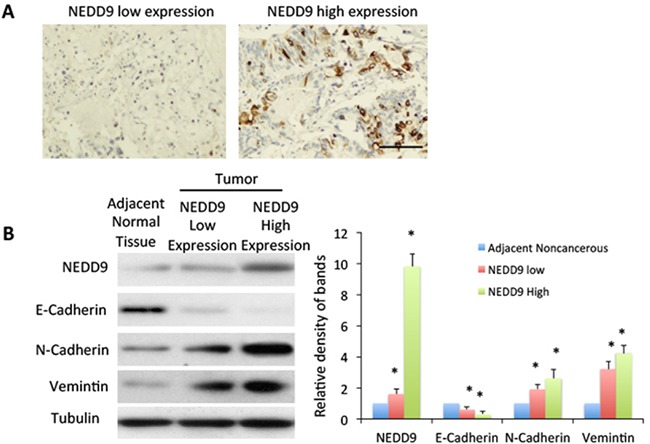
High expression of NEDD9 related to epithelial-mesenchymal transition **A.** Expression of NEDD9 in human hepatocellular carcinoma tissue. Tumors were collected and immunohistochemistry used to test NEDD9 expression. Scale bar, 100 μm. **B.** Equal amounts of cell lysate from tumor tissues and paired normal tissues from patients analyzed by western blot for indicated proteins. Band density was quantified. * p <0.01 compared to adjacent normal tissue.

We collected human HCC tissues with high expression of NEDD9 (Figure 1A, 1B) and tested the expression of epithelial marker E-cadherin and mesenchymal markers N-cadherin and vimentin. Compared to adjacent normal tissue, HCC tissues with high NEDD9 expression exhibited significant downregulation of E-cadherin and dramatic upregulation of N-cadherin and vimentin in comparison to adjacent normal tissue (Figure 1B). This upregulation indicated the activation of EMT in NEDD9-overexpressing HCC tissues.

### NEDD9 regulated metastasis of HCC cells

**Figure 2:**
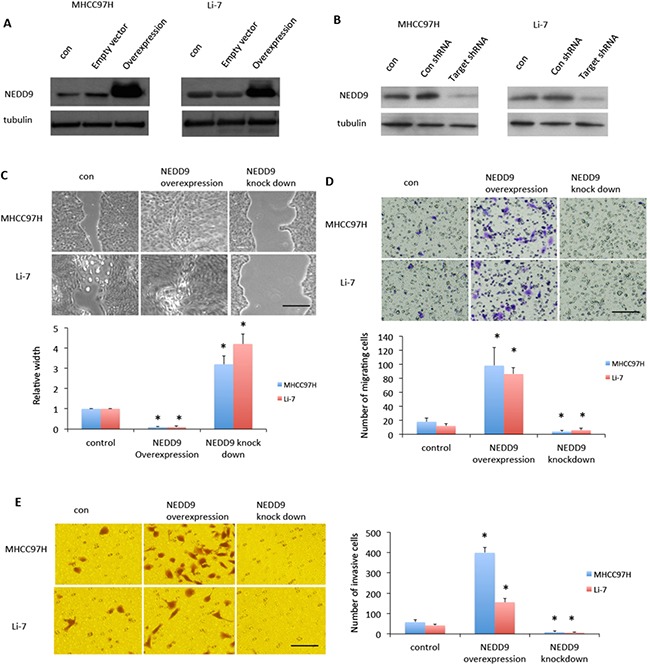
NEDD9 regulated hepatocellular carcinoma cells migration and invasion NEDD9 was either **A.** overexpressed or **B.** knocked down in MHC97H or Li-7 hepatocellular carcinoma (HCC) cells. **C.** Wound healing assays tested migration ability of HCC cells. After 48 h, images were taken and representative images are shown. **D.** Transwell assays were used to test test migration ability of HCC cells. After 48 h, images were taken and representative images are shown. **E.** Matrigel invasion assays were used to test migration ability of HCC cells. After 24 h, images were taken and representative images are shown. Scale bar, 50 μm.* p <0.01 compared to control cells.

Since EMT promotes malignant cell invasion into the tissues surrounding most carcinomas [16], we overexpressed or suppressed NEDD9 in HCC cell lines MHCC97H and Li-7, which have a highly aggressive phenotype (data not shown) (Figure 2A, 2B). We next used wound-healing assay and matrigel invasion assay to test metastatic capacity of HCC cells. After cells were incubated with serum-free DMEM overnight, MTT assay showed HCC cells stopped proliferation over the next 48h (Supplementary Figure S2). We then used the starved cells for following experiments. Wound-healing assays showed that NEDD9-overexpressing cells migrated faster than control cells and NEDD9-repressed cells migrated slower than control cells (Figure 2C, 2D).

**Figure 3:**
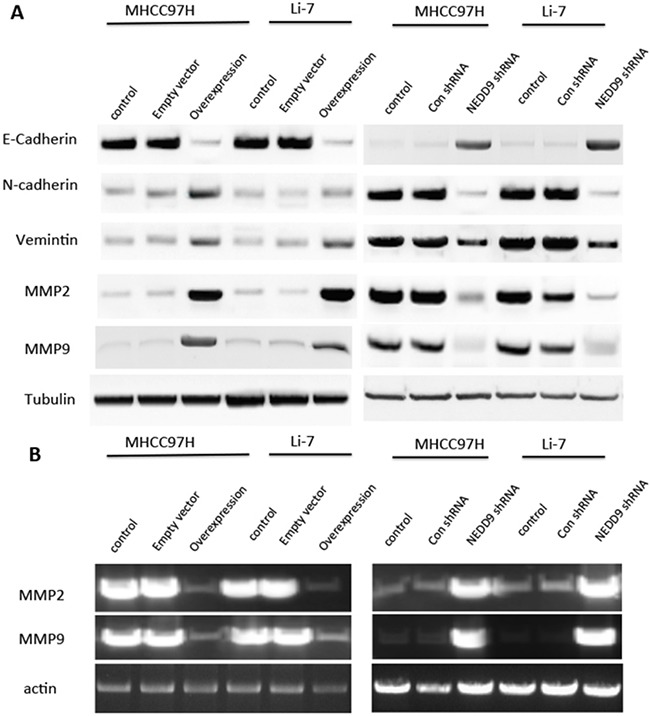
NEDD9 regulated epithelial-mesenchymal transition markers and matrix metalloproteinases in hepatocellular carcinoma cells **A.** Cell lysates were collected and western blots used to test EMT markers E-cadherin, N-cadherin and vimentin and MMP2 and MMP9 expression in hepatocellular carcinoma cells. **B.** The activities of MMP2 and MMP9 were determined by gelatinase zymography with cell supernatant.

Matrigel invasion assays showed that NEDD9-overexpressing cells had faster invasion than control cells and NEDD9-repressed cells had slower invasion than control cells (Figure 2E). We also tested EMT markers in the HCC cell lines MHCC97H and Li-7 (Figure 3). E-cadherin expression was downregulated in NEDD9-overexpressing cells and upregulated in NEDD9-repressed cells. N-cadherin and vimentin expression was elevated in NEDD9-overexpressing cells and repressed in NEDD9-repressing cells (Figure 3). Matrix metalloproteinases (MMPs), which are secreted by all cancer cells, are required to cleave cell adhesion molecules such as E-cadherin and degrade extracellular matrix (ECM) proteins. In this way, MMPs are responsible for the metastasis of cancer cell [17]. In this study, MMP2 and MMP9 expression and activity were higher when NEDD9 was overexpressed and lower when NEDD9 was depleted (Figure 3A, 3B).

### NEDD9 modulates CSC properties in HCC cells

**Figure 4:**
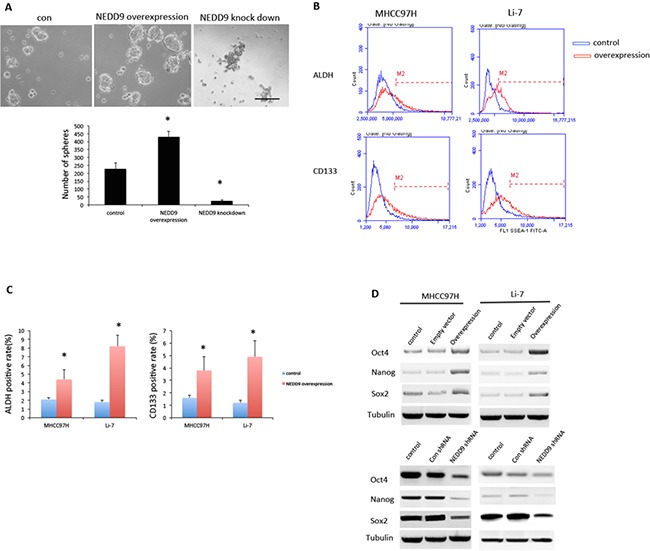
NEDD9 regulated cancer stem cell phenotype in hepatocellular carcinoma cells **A.** Sphere formation assays were used to test sphere-forming abilities of MHCC97H cells. Scale bar, 100 μm. **B** and **C.** Fluorescence-activated cell sorting analysis showed increased cancer stem cell (CSC) markers ALDH, and CD133 in NEDD9-overexpressing MHCC97H cells. **D.** Western blots were used to test indicated CSC markers Oct-2, Nanog and Sox2 in hepatocellular carcinoma cells. * p <0.01 compared to control cells.

EMT is related to stem-like properties in cancer cells. We assessed if NEDD9 modulated the CSC properties by examined the sphere-forming ability of MHCC97H cells. Overexpression of NEDD9 increased the number of spheres formed by HCC cells. Loss of NEDD9 reduced the number of spheres made by HCC cells (Figure 4A).

Using flow cytometry, we found that the CSC markers of aldehyde dehydrogenase (ALDH) activity and CD133 expression were significantly higher in NEDD9-overexpressing HCC cells (Figure 4B, 4C). ALDH activity increased 1-fold in MHCC97H and 4.4-fold Li-7 cells, and CD133 expression increased 2.4-fold in MHCC97H and 4.1-fold in Li-7 cells compared to control cells that transfected by empty vectors. Stem cell markers Nanog, Oct4 and Sox2 were upregulated in NEDD9-overexpressing HCC cells and downregulated when NEDD9 was knocked down (Figure 4D).

### NEDD9 modulated CSCs phenotypes through regulation of Smad7

**Figure 5:**
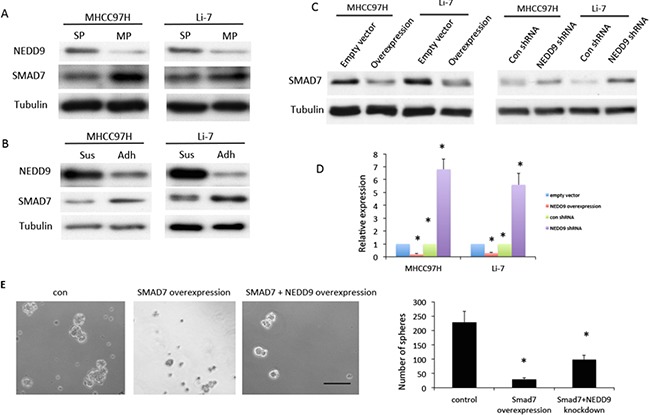
NEDD9 regulated cancer stem cell phenotype through Smad7 in hepatocellular carcinoma cells Western blots were used to test **A.** expression of NEDD9 and Smad7 in a side population (SP) and main population (MP) isolated by fluorescence-activated cell sorting. **B.** Expression of NEDD9 and Smad7 in suspension (Sus) and attached (Adh) hepatocellular carcinoma (HCC) cells. **C.** Expression of Smad7 in NEDD9-overexpressing and NEDD9-knockdown HCC cells. **D.** Quantitative PCR was used to test mRNA of Smad7 in NEDD9-overexpressing and NEDD9-depleted HCC cells. **E.** Sphere-formation assays were used to test sphere-forming abilities of NEDD9-overexpressing and NEDD9-depleted HCC cells. Scale bar, 100 μm. * p <0.01 compared to control cells.

To explore the possible mechanism by which NEDD9 modulated EMT and stemness, the effects of NEDD9 on the Smad pathway was examined. Compared with relevant controls, Smad7 expression decreased in sphere cells and isolated stem-like cells (Figure 5A, 5B). Smad7 expression at both the mRNA and protein levels was downregulated in NEDD9-overexpressing HCC cells and upregulated in NEDD9-depleted HCC cells (Figure 5C, 5D). This deregulation may result in activation of Smad signaling.

We examined the effects of Smad7 on sphere-formation ability as regulated by NEDD9. Overexpression of Smad7 inhibited sphere formation of HCC cells and partially blocked the increase in sphere formation induced by overexpression of NEDD9 (Figure 5E).

### NEDD9 bound the FAK-Src-Crk complex and modulated cell-extracellular matrix adhesion

**Figure 6:**
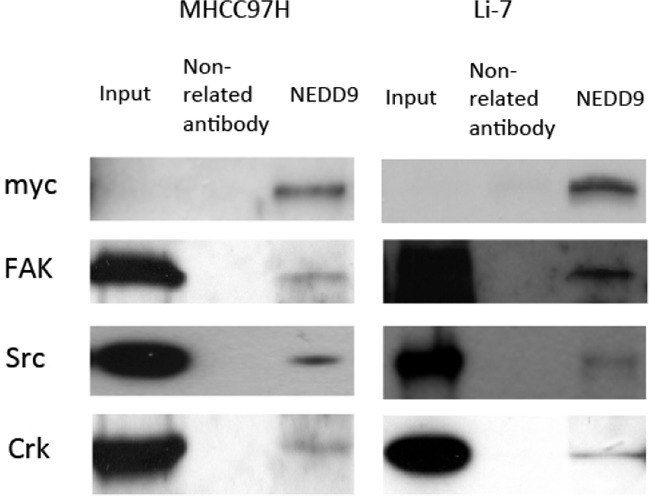
NEDD9 binded to FAK-Src-Crk complex NEDD9 was transfected into hepatocellular carcinoma cells. Immunoprecipitation was used to test indicated proteins in NEDD9-overexpressing hepatocellular carcinoma cells. Protein was precipitated by anti-NEDD9, and western blots were used to test for Fak, Src and Crk protein.

**Figure 7:**
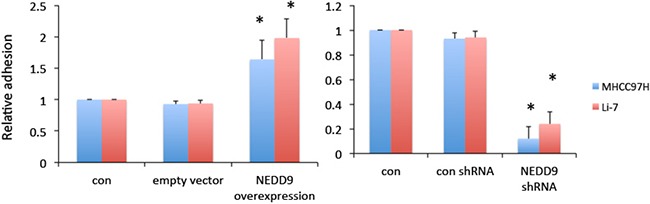
NEDD9 enhanced cell-to-extracellular matrix adhesion Hepatocellular carcinoma control, NEDD9-overexpressing, or NEDD9-knockdown cells were plated into 96-well plates coated with fibronectin and incubated at 37°C for 1 h. Cell adhesion was measured using an ELISA reader after staining with 0.1% crystal violet. * p <0.01 compared to control cells.

The Cas-Crk complex has been described as a master switch for cell migration [22]. We tested if moderate overexpression of NEDD9 drove the assembly of functional Cas-Crk complexes. Exogenous NEDD9 was transfected into HCC cells and immunoprecipitation showed that NEDD9 bound to FAK, Src and Crk (Figure 6). NEDD9 is an important component of integrin receptor signaling that is crucial for cell-to-ECM adhesion and migration. Overexpression of NEDD9 significantly increased the adhesion of HCC cells and loss of NEDD9 reduced adhesion (Figure 7).

### NEDD9 is crucial for HCC metastasis in a xenograft model

A lung metastasis model of HCC was used to determine the effect of NEDD9 on tumor metastasis *in vivo*. At 6 weeks after injection of HCC cells, sacrificed mice were dissected to investigate visible metastatic lesions in livers (Table 1). Of 10 mice that received MHCC97H cells, 5 had lung metastasis, while metastasis was seen in 6 of 10 mice that received Li-7 cells. All of mice injected with NEDD9-overexpressing HCC cells had metastasis to the lungs. Of 10 mice receiving HCC cells with NEDD9 deleted, only 2 or fewer showed metastasis.

**Table 1:** Lung metastasis of HCC cells *in vivo* (n = 10 mice)

	con	empty vector	NEDD9 overexpression	con shRNA	NEDD9 shRNA
MHCC97H	5	5	10	6	2
Li-7	6	6	10	5	1

con, control; HCC, hepatocellular carcinoma; shRNA,

## DISCUSSION

This study demonstrated that NEDD9 was crucial for migration and invasion of HCC cells *in vitro* and for metastasis *in vivo*. This finding might be explained by reduced cell-to-cell adhesion, higher ability to separate cells from the ECM and enhanced adhesion of cells to the ECM at metastasis sites [27]. NEDD9-overexpressing HCC cells were positive for EMT markers. Fewer of these markers were found in NEDD9-repressed HCC cells, suggesting that NEDD9 was a key EMT factor in HCC cells, possibly resulting in the impairment of cell-to-cell adhesion, leading to high migratory ability.

Tikhmyanova found that in human mammary epithelial cells, NEDD9 promotes removal of E-cadherin from the plasma membrane and lysosomal degradation [28]. Cancer cells secrete MMPs such as MMP2 and MMP9 to promote invasion into surrounding tissues. HCC cells with upregulated NEDD9 expressed more MMP2 and MMP9, potentially increasing their invasive ability. Cell-to-ECM adhesion is another step by which cancer cells invade remote tissue [29]. We found that this adhesion was enhanced when NEDD9 was upregulated and repressed when NEDD9 was downregulated. These results provide a possible reason that NEDD9 promoted HCC metastasis.

CSCs are involved in metastasis. At the invading front of pancreatic tumors, a distinct subpopulation of CD133+CXCR4+ cancer stem cells determines the metastatic phenotype of individual tumors [30]. When pancreatic carcinoma cells are depleted of CD133+CXCR4+, the metastatic potential of cancer cells is abrogated without affecting their tumorigenic potential. This result suggests that a subset of CSCs is responsible for metastasis. Tumorigenic CD44+CD24−/low cells are also detected in metastatic pleural fluid in breast cancer patients and breast CSC phenotypes are found in bone marrow [31]. In this study, NEDD9-overexpressing HCC cells had more ALDH-positive and CD133-positive cells compared to control nonoverexpressing cells. CSCs might lead to both the EMT and faster metastasis. However, how NEDD9 regulates the properties and fate of CSCs is not clear. NEDD9 repressed Smad7 expression at both the transcriptional and post-transcriptional levels to activate Smad signaling and induce EMT and adhesion. Overexpression of Smad7 impaired sphere formation in HCC cells, indicating its importance in the fate of CSCs.

In our study, NEDD9 bound to the FAK-Src-Crk complex, consistent with the finding that NEDD9 positively regulates the FAK-Src-Crk migratory switch [22]. Initial reports identifying NEDD9 established that it directly interacts with FAK, Src, and Crk [34]. These interactions enhance the activation of Src and FAK and lead to extensive tyrosine phosphorylation of NEDD9. This modification creates binding sites for effector proteins with SH2 domains, including Crk and Crk-L. The Cas-Crk complex is described as a master switch for cell migration. By interacting with Crk-L and DOCK180, NEDD9 activates Rac and other components of the cell-migration machinery [22, 35].

In conclusion, NEDD9 was crucial for metastasis of HCC cells. It downregulated Smad7, resulting in activation of Smad signaling. It binded FAK-Src-Crk complex to promote EMT and stemness in HCC cells.

## MATERIALS AND METHODS

### Patients and tumor specimens

Human HCC specimens were obtained from patients with a diagnosis of HCC at Xijing hospital, Xi'an, China. The diagnosis of HCC and the pathological grade were confirmed by histological examination. No patients had undergone preoperative radiotherapy or chemotherapy. All samples were collected immediately after surgery and stored in formalin and embedded into paraffin.

### Cell culture and gene transfection

MHCC97H and Li-7 human HCC cell lines were obtained from the Chinese Academy of Science (Shanghai, China). MHCC97H and Li-7 cells were maintained on plates at 37°C, 5% CO_2_ in Dulbecco's Modified Eagle Medium (DMEM) (GIBCO) and Roswell Park Memorial Institute (RPMI) 1640 medium (Thermo Fisher Scientific Co.), respectively, supplemented with 10% FBS, 100 units/ml penicillin and 0.1 mg/ml streptomycin. For gene transfection, cell lines were cultured overnight in 6-well plates (1.0 × 10^6^ cells/well) and transfected with 5 μg/ml plasmid pcDNA5/FRT/TO-neo-Myc-NEDD9 (Lot No. O11265) (Genepharma, Shanghai, China.), or empty plasmid using 5 μl Lipofectamine 2000 reagent (Invitrogen, USA). Cells were incubated for 24 h and positive clones were selected using 600 μg/ml G418 (Sigma, USA) selection. For knock down assay, cells were transfected with 10μg/ml plasmid pLKO.1 puro-NEDD9-shRNA(Lot No. A11265) (Genepharma, Shanghai, China.), or empty plasmid using 5 μl Lipofectamine 2000 reagent (Invitrogen, USA). Cells were incubated for 24 h and positive clones were selected using 100 μg/ml puromycin (Sigma, USA) selection.

### Immunohistochemistry

Paraffin-embedded sections of human tumor tissues were stained according to standard protocols with primary antibody against NEDD9 (1:200 dilution, Abcam, USA) followed by staining with biotinylated anti-mouse secondary antibody (1:500 dilution, Abcam, USA). After washing to remove unbound antibodies, sections were incubated with buffer containing horseradish peroxidase-conjugated streptavidin followed by addition of substrate solution containing peroxidase. Slides were mounted with 3,3′-diaminobenzidine and visualized with a Leica lightfield microscope.

### Western blots

Western blots were performed according to a standard protocol [24] using cell or tumor tissue lysates. Proteins were resolved by SDS-PAGE and transferred to polyvinylidene fluoride membranes. Indicated antibodies were applied overnight at 4°C. Anti-E-cadherin, anti-N-cadherin, anti-vimentin, anti-Sox2, anti-Oct4 and anti-Nanog were from Cell Signaling (USA) (all 1:1000 dilution). Anti-tubulin antibody was from Sigma (USA) (1:5000 dilution). Blots were incubated with secondary horseradish peroxidase-conjugated IgG (1:10,000 dilution, Cell Signaling, USA) and visualized with enhanced chemiluminescence reagents. Experiments were repeated three times.

### Immunoprecipitation

Cell lysates were incubated with control antibody or anti-NEDD9 overnight at 4°C with gentle vortexing. Protein-G agarose resin was added for 3 h at 4°C. After centrifugation, protein-G agarose was washed five times with washing buffer. Loading buffer was added and samples were boiled for 5 min and analyzed with anti-Fak, anti-Src, or anti-Crk.

### Real-time PCR

Cells were lysed using TRIzol reagent (TIANGEN, Beijing, China) and total RNA was isolated according to the manufacturer's protocol. Isolated RNA was reverse-transcribed into cDNA using SuperScript II Reverse Transcriptase (Invitrogen, USA). Gene expression was measured using iQSYBR Green Supermix (Bio-Rad, USA) in a Bio-Rad iQ5 thermal cycling system (Bio-Rad, USA) following supplier instructions. Primer sequences were: *Smad7* **F**: 5′-AGGTGTTCCCCGGTTTCTCCA-3′, **R**: 5′-TTCACAAAGCTGATCTGCACGGT-3′; *GAPDH* **F** 5′-CAAGGTCATCCATGACAACTTCG-3′ **R** 5′-GTCC ACCACCCTGTTGCTGTAG-3′.

### Gelatin zymography

Cells were cultured in SFCM for 24 hrs. The cultured medium supernatant was harvested and incubated with Gelatin Sepharose 4B beads shaking for 10h at 4°C. The extract samples were then subjected to electrophoresis (150 V for 3 h) on 10% SDS-PAGE (sodium dodecyl sulfate polyacrylamide gel electrophoresis) containing 0.1% gelatin. After electrophoresis, gels were washed in 2.5% Triton-X-100 at room temperature and then incubated overnight in reaction buffer (40 mM Tris–HCl, pH 8.0; 10 mM CaCl2, and 0.02 % NaN3) at 37°C overnight. After incubation, activities of MMPs were determined by analyzing signal intensity using Gel Pro v.4.0 software (Media Cybernetics, Silver Spring, MD, USA).

### Flow cytometry for CD133 expression and ALDH activity

Cells (2 × 10^5^) were counted and incubated in flow buffer (2% FBS in PBS) containing CD133 antibody (BD Biosciences, USA) for 20 min at 4°C. Unbound antibodies were washed with flow buffer and cells were analyzed using a FACS SCAN (BD Biosciences, USA). ALDEFLUOR kits (StemCell Technologies, USA) were used to determine increased ALDH enzymatic activity. Cells (2 × 10^5^) cells were suspended in ALDEFLUOR assay buffer containing ALDH substrate and incubated for 30 min at 37°C. Intracellular fluorescence was measured by flow cytometry using a FACS SCAN and Cell Quest ProTM software. All experiments were performed in triplicate and repeated three times.

### Wound healing assays

Cell migration was analyzed by wound healing assays. Cells were grown to confluence and wounds generated by dragging a 200-μl pipette tip across the monolayer. Cells were cultured as described above, and wound closure was followed by microscopy at 48 h after wounding. Experiments were repeated three times.

### Transwell assays

Cell migration was analyzed by Transwell assays. Cells (3 × 10^4^) in serum-free DMEM were seeded into upper chambers of 8-μM-pore Transwells and allowed to migrate for 12 h. Migrated cells were fixed and stained with 0.1% crystal violet (Wako Pure Chemical Industries, Ltd., Osaka, Japan), counted from six random fields and averaged. Experiments were repeated three times.

### Invasion assays

Cells (3 × 10^4^) in serum-free DMEM were seeded into upper chambers of 8-μM-pore Transwells with a Matrigel barrier and allowed to migrate for 24 h. Migrated cells were fixed and stained with 0.1% crystal violet, counted from six random fields and averaged. Experiments were repeated three times.

### Cell adhesion assays

Assays used 96-well plates coated with fibronectin at 1 μg/well (Sigma) and dried at room temperature. Cells were plated into each well and incubated at 37°C for 1 h. PBS was added to remove nonadherent cells. Adhesive cells were fixed with 3% formaldehyde and stained with 0.1% crystal violet. After washing with PBS, 10% acetic acid was added, and absorbance at 570 nm was determined using an ELISA plate reader (iMark Microplate Reader; Bio-Rad, Hercules, CA, USA). Experiments were repeated three times.

### Tumorsphere formation assays

Ability of clones to form floating tumorspheres in nonadhesive conditions was evaluated by plating about 1 × 10^3^ cells per well in 24-well ultralow attachment plates (Corning Inc., Corning, NY, USA) in serum-free DMEM/F12 supplemented with 2% B27 and 1% N2 supplement (Life Technologies, Invitrogen, USA), 20 ng/ml human epidermal growth factor, 10 ng/ml human basic fibroblast growth factor and 5μg/ml insulin. After 14 days at 37°C, plates were observed for tumorsphere formation. Spheres were dissociated using a nonenzymatic solution (Sigma, USA) and replated at 1000 cells per well for another 14 days. The number and size of spheres were quantified using an inverted microscope. Experiments were repeated three times.

### *In vivo* metastasis studies

Male athymic nude mice (4 weeks old) were from the Experimental Animal Center of the Fourth Military Medical University. All animal procedures were performed in accordance with protocols approved by the Animal Care and Use Committee of the Fourth Military Medical University. Mice were injected through the tail vein with 5 × 10^6^ cells, either MHCC97H controls, MHCC97H cells transfected with empty vector, NEDD9-overexpressing MHCC97H cells or NEDD9-knockdown MHCC97H cells. Mice were sacrificed at day 42 after injection and lungs were inspected for tumor formation.

### Statistical analysis

Statistical significance of treatment outcomes between different groups was assessed using the one way ANOVA and p < 0.05 was considered statistically significant for all analyses.

## SUPPLEMENTARY FIGURES



## Abbreviations

NEDD9: developmentally downregulated 9
HCC: hepatocellular carcinoma
EMT: epithelial-mesenchymal transition
MMP2: matrix metalloprotein 2
ALDH: aldehyde dehydrogenase
CSCs: cancer stem cells
ECM: extracellular matrix.

## References

[1] Tang ZY, Ye SL, Liu YK, Qin LX, Sun HC, Ye QH, Wang L, Zhou J, Qiu SJ, Li Y, Ji XN, Liu H, Xia JL, Wu ZQ, Fan J, Ma ZC, Zhou XD, Lin ZY, Liu KD (2004). A decade's studies on metastasis of hepatocellular carcinoma. J Cancer Res Clin Oncol.

[2] Min J, Liu L, Li X, Jiang J, Wang J, Zhang B, Cao D, Yu D, Tao D, Hu J, Gong J, Xie D (2015). Absence of DAB2IP promotes cancer stem cell like signatures and indicates poor survival outcome in colorectal cancer. Sci Rep.

[3] Kalluri R, Weinberg RA (2009). The basics of epithelial-mesenchymal transition. J Clin Invest.

[4] Hanahan D, Weinberg RA (2011). Hallmarks of cancer: the next generation. Cell.

[5] Mani SA, Guo W, Liao MJ, Eaton EN, Ayyanan A, Zhou AY, Brooks M, Reinhard F, Zhang CC, Shipitsin M, Campbell LL, Polyak K, Brisken C, Yang J, Weinberg RA (2008). The epithelial-mesenchymal transition generates cells with properties of stem cells. Cell.

[6] Thiery JP, Sleeman JP (2006). Complex networks orchestrate epithelial-mesenchymal transitions. Nat Rev Mol Cell Biol.

[7] Minegishi M, Tachibana K, Sato T, Iwata S, Nojima Y, Morimoto C (1996). Structure and function of cas-l, a 105-kd crk-associated substrate-related protein that is involved in beta 1 integrin-mediated signaling in lymphocytes. J Exp Med.

[8] Law SF, Estojak J, Wang B, Mysliwiec T, Kruh G, Golemis EA (1996). Human enhancer of filamentation 1, a novel p130cas-like docking protein, associates with focal adhesion kinase and induces pseudohyphal growth in saccharomyces cerevisiae. Mol Cell Biol.

[9] Kumar S, Tomooka Y, Noda M (1992). Identification of a set of genes with developmentally down-regulated expression in the mouse brain. Biochem Biophys Res Commun.

[10] Tikhmyanova N, Little JL, Golemis EA (2010). Cas proteins in normal and pathological cell growth control. Cell Mol Life Sci.

[11] Minn AJ, Gupta GP, Siegel PM, Bos PD, Shu W, Giri DD, Viale A, Olshen AB, Gerald WL, Massague J (2005). Genes that mediate breast cancer metastasis to lung. Nature.

[12] Natarajan M, Stewart JE, Golemis EA, Pugacheva EN, Alexandropoulos K, Cox BD, Wang W, Grammer JR, Gladson CL (2006). Hef1 is a necessary and specific downstream effector of fak that promotes the migration of glioblastoma cells. Oncogene.

[13] Kim M, Gans JD, Nogueira C, Wang A, Paik JH, Feng B, Brennan C, Hahn WC, Cordon-Cardo C, Wagner SN, Flotte TJ, Duncan LM, Granter SR, Chin L (2006). Comparative oncogenomics identifies nedd9 as a melanoma metastasis gene. Cell.

[14] Lu P, Wang ZP, Dang Z, Zheng ZG, Li X, Zhou L, Ding R, Yue SQ, Dou KF (2015). Expression of NEDD9 in hepatocellular carcinoma and its clinical significance. Oncol Rep.

[15] Qin Y, Tang B, Hu CJ, Xiao YF, Xie R, Yong X, Wu YY, Dong H, Yang SM (2016). An hTERT/ZEB1 complex directly regulates E-cadherin to promote epithelial-to-mesenchymal transition (EMT) in colorectal cancer. Oncotarget.

[16] Polyak K, Weinberg RA (2009). Transitions between epithelial and mesenchymal states: acquisition of malignant and stem cell traits. Nat Rev Cancer.

[17] van Zij F, Krupitza G, Mikulits W (2011). Initial Steps of Metastasis: Cell Invasion and Endothelial Transmigration. Mutat. Res.

[18] Leng A, Liu T, He Y, Li Q, Zhang G (2009). Smad4/Smad7 balance: A role of tumorigenesis in gastric cancer. Exp Mol Pathol.

[19] Lan HY, Chung AC (2011). Transforming growth factor-β and Smads. Contrib Nephrol.

[20] Huang Q, Liu L, Liu CH, Shao F, Xie F, Zhang CH, Hu SY (2012). Expression of Smad7 in cholangiocarcinoma: prognostic significance and implications for tumor metastasis. Asian Pac J Cancer Prev.

[21] Javelaud D, Mohammad KS, McKenna CR (2007). Stable overexpression of smad7 in human melanoma cells impairs bone metastasis. Cancer Res.

[22] O'Neill GM, Fashena SJ, Golemis EA (2000). Integrin signaling: a new Cas(t) of characters enters the stage. Trends Cell Biol.

[23] Law SF, Estojak J, Wang B, Mysliwiec T, Kruh G, Golemis EA (1996). Human enhancer of filamentation 1, a novel p130cas-like docking protein, associates with focal adhesion kinase and induces pseudohyphal growth in saccharomyces cerevisiae. Mol Cell Biol.

[24] Astier A, Manie SN, Law SF, Canty T, Haghayghi N, Druker BJ, Salgia R, Golemis EA, Freedman AS (1997). Association of the cas-like molecule hef1 with crkl following integrin and antigen receptor signaling in human b-cells: potential relevance to neoplastic lymphohematopoietic cells. Leuk Lymphoma.

[25] Lucas JJ, Salimath BP, Slomiany MG, Rosenzweig SA (2010). Regulation of invasive behavior by vascular endothelial growth factor is hef1-dependent. Oncogene.

[26] Zhang SS, Wu LH, Liu Q, Chen KS, Zhang XF (2015). Elevated expression of NEDD9 is associated with metastatic activity in gastric cancer. Onco Targets Ther.

[27] Lin YN, Bhuwania R, Gromova K, Failla AV, Lange T, Riecken K, Linder S, Kneussel M, Izbicki JR, Windhorst S (2015). Drosophila homologue of Diaphanous 1 (DIAPH1) controls the metastatic potential of colon cancer cells by regulating microtubule-dependent adhesion. Oncotarget.

[28] Tikhmyanova N, Golemis EA (2011). NEDD9 and BCAR1 negatively regulate E-cadherin membrane localization, and promote E-cadherin degradation. PLoS One.

[29] Olsen JR, Oyan AM, Rostad K, Hellem MR, Liu J, Li L, Micklem DR, Haugen H, Lorens JB, Rotter V, Ke XS, Lin B, Kalland KH (2013). p63 attenuates epithelial to mesenchymal potential in an experimental prostate cell model. PLoS One.

[30] Hermann PC, Huber SL, Herrler T, Aicher A, Ellwart JW, Guba M, Bruns CJ, Heeschen C (2007). Distinct populations of cancer stem cells determine tumor growth and metastatic activity in human pancreatic cancer. Cell Stem Cell.

[31] Balic M, Lin H, Young L, Hawes D, Giuliano A, McNamara G, Datar RH, Cote RJ (2006). Most early disseminated cancer cells detected in bone marrow of breast cancer patients have a putative breast cancer stem cell phenotype. Clin Cancer Res.

[32] Katsuno Y, Lamouille S, Derynck R (2013). Tgf-beta signaling and epithelial-mesenchymal transition in cancer progression. Curr Opin Oncol.

[33] Izumchenko E, Singh MK, Plotnikova OV, Tikhmyanova N, Little JL, Serebriiskii IG, Seo S, Kurokawa M, Egleston BL, Klein-Szanto A, Pugacheva EN, Hardy RR, Wolfson M, Connolly DC, Golemis EA (2009). NEDD9 promotes oncogenic signaling in mammary tumor development. Cancer Res.

[34] Martin-Rendon E, Hale SJ, Ryan D, Baban D, Forde SP, Roubelakis M, Sweeney D, Moukayed M, Harris AL, Davies K, Watt SM (2007). Transcriptional profiling of human cord blood CD133+ and cultured bone marrow mesenchymal stem cells in response to hypoxia. Stem Cells.

[35] O'Neill GM1, Seo S, Serebriiskii IG, Lessin SR, Golemis EA (2007). A new central scaffold for metastasis: parsing HEF1/Cas-L/NEDD9. Cancer Res.

