# Supplementary material for: NEDD9 may regulate hepatocellular carcinoma cell metastasis by promoting epithelial-mesenchymal-transition and stemness via repressing Smad7
Source: Oncotarget. 2016 Dec 10;8(1):1714–24. doi: 10.18632/oncotarget.13852 (PMC5352091; doi:10.18632/oncotarget.13852)
Supplement: Supplementary file 1 [file oncotarget-08-1714-s001.pdf]

NEDD9 may regulate hepatocellular carcinoma cell metastasis by promoting epithelial-mesenchymal-transition and stemness via repressing Smad7

SUPPLEMENTARY FIGURES

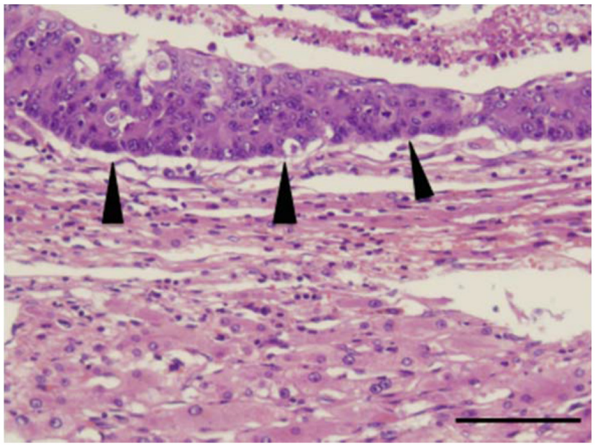

Supplementary Figure S1: HE staining for Human HCC samples. Human HCC samples were collected and fixed and subjected to HE staining. Representative image showed the HCC tissue and adjacent normal liver tissue. Arrows showed the HCC tissue. Scale bar, 100 μm.

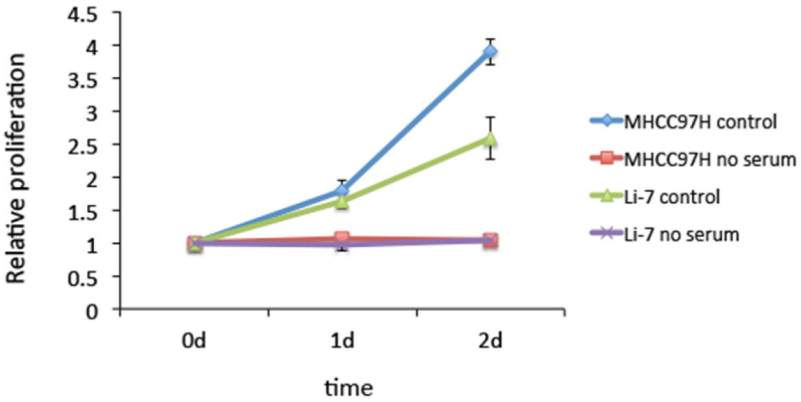

Supplementary Figure S2: HCC cells were cultured overnight in serum-free condition. The proliferation was tested at 0d, 1d or 2d by using MTT.

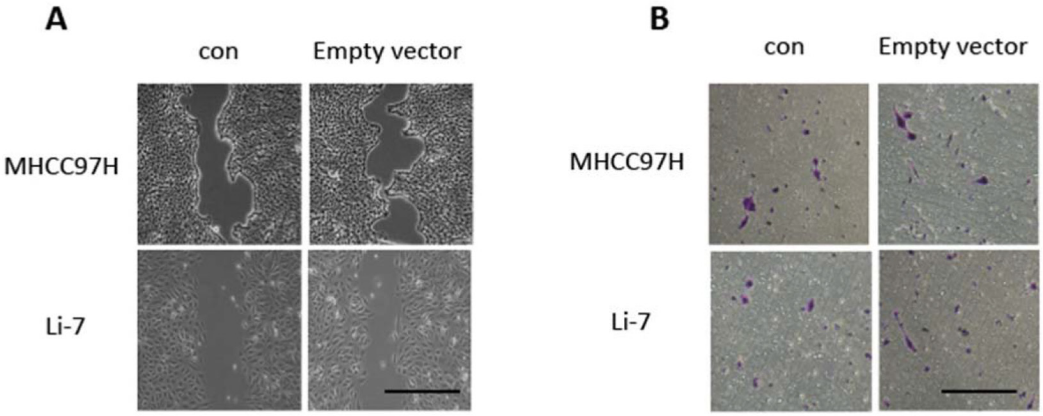

Supplementary Figure S3: HCC cells were transfected with empty vector. A. Wound healing assays and Transwell assays **B.** were used to test migration ability of HCC cells. After 48 h, images were taken and representative images are shown. Scale bar, 50 μm.
